# Perirenal Hematoma: A Rare Complication of Anticoagulant Therapy

**DOI:** 10.7759/cureus.48986

**Published:** 2023-11-18

**Authors:** Frederico Silva, Joana Moutinho, Joana Nascimento, Tiago Vasconcelos, Inês G Simões

**Affiliations:** 1 Internal Medicine, Centro Hospitalar Universitário do Algarve - Unidade de Portimão, Portimão, PRT

**Keywords:** diagnostic imaging, hemorrhagic complications, complications of anticoagulation, enoxaparin, perirenal hematoma

## Abstract

Hemorrhagic complications arising from anticoagulant use are a well-recognized concern in clinical practice. This case study presents an 84-year-old woman with multiple cardiovascular risk factors, including atrial fibrillation, who developed a perirenal hematoma after just five doses of enoxaparin, prescribed for stroke prevention. The patient exhibited altered mental status and abdominal pain, prompting imaging studies revealing the hematoma. This case highlights the importance of vigilance in patients at risk for bleeding complications, especially in the initial days of anticoagulant therapy. Diagnostic imaging, particularly CT scans or ultrasound, is crucial for early detection. Management strategies range from discontinuing anticoagulants to potential interventions like anticoagulation reversal, angiography, or surgery. The decision to resume anticoagulation presents a challenge and requires a personalized approach based on patient factors. This case underscores the need for continued vigilance, early diagnosis, and evidence-based decisions in managing patients on anticoagulants, emphasizing the necessity for further research to establish clear guidelines in such complex clinical scenarios.

## Introduction

Hemorrhagic complications arising from anticoagulant use are widely recognized, along with their associated risk factors. Striking a balance between risk and benefit is crucial for every patient and situation. Among these infrequent complications is perirenal hematoma, a condition with potentially fatal outcomes and various management approaches.

## Case presentation

An 84-year-old woman arrived at the emergency department exhibiting subacute changes in mental status and aggressive behavior directed toward her family members. Her past medical history included hypertension, persistent atrial fibrillation, heart failure, dyslipidemia, and a history of cerebrovascular disease with a previous stroke that did not result in significant aftereffects. The patient's regular medications included: losartan 50 mg once daily, carvedilol 6.25 mg once daily, furosemide 40 mg once daily, rosuvastatin 20 mg once daily, apixaban 2.5 mg twice daily (dose reduction of apixaban due to age over 80 years old and baseline creatinine level of 1.6 mg/dL). Upon symptom review, it was noted that she had been experiencing behavioral changes and fluctuating altered mental status over the past three weeks. At first, there was worry about a potential stroke given the patient's medical history and anamnestic findings related to cardiovascular risk factors. Upon admission, she reported no fever, nausea, vomiting, abdominal pain, or respiratory symptoms. The patient acknowledged experiencing considerable confusion in recent weeks. Additionally, she mentioned forgetting to take her medication and having strained relationships with her two daughters due to their persistent efforts to place her in a nursing home.

On physical examination, the patient was alert, conscious, and oriented in person and space, but not in time. The patient's speech was polite, appropriate, and somewhat verbose. Amnesia for recent events. No motor or sensitive deficits. Normal facial expression and tongue protrusion in the midline. No dysarthria or aphasia. Pupils were equal in size and reaction to light, centered. No double vision or visual field deficits. Plantar cutaneous reflex was indifferent bilaterally. Mucous membranes were pink and moist, non-jaundiced. Breathing comfortably on room air without signs of respiratory distress. Pulmonary auscultation reveals a maintained symmetrical vesicular murmur. Cardiac sounds were irregular, without audible murmurs. The abdomen was soft and depressible, non-tender on palpation, without masses or enlarged lymph nodes. No edema in the lower extremities, no signs of deep vein thrombosis. Elevated blood pressure was observed at 175/97 mmHg, accompanied by an irregular heart rate of 80 beats per minute. The patient's temperature was measured at 36.6ºC. The National Institutes of Health Stroke Scale (NIHSS) score was recorded as 3 (2 points for not answering both questions correctly, that is month and age, and 1 point for performing only one task correctly, that is "blink eyes & squeeze hands").

The brain CT scan revealed a hypodensity and slight expansion in the left pre-central inferior cortical area, indicating a potential recent infarction. Additionally, scattered hypodensities were observed in the junctional/frontal, subcortical, and radiating regions of both cerebral hemispheres, likely due to microangiopathic causes and potentially associated with significant carotid axis stenosis. Evidence of likely infarction sequelae was found in the superior right external parietal cortico-subcortical cavity. Furthermore, cavitations were present in the inferior and posterior cerebellar folia on the right side, suggesting possible arteriolar infarction sequelae.

Psychiatry was consulted, who found too many organic causes for the clinical scenario.

The blood test results revealed no abnormalities regarding evidence of infection, anemia, dyslipidemia, renal failure, or electrolyte imbalances. The international normalized ratio (INR) was 0.9 (normal value < 1.1) and activated partial thromboplastin time (APTT) was 35.8 seconds (normal range 30 - 40).

The patient was subsequently admitted for further evaluation of altered mental status and to investigate the likely cause of the recent stroke. Considering the patient's low NIHSS score and uncertain timeline of the stroke, anticoagulation therapy was started with enoxaparin 60 mg every 12 hours. Anticoagulation was initiated upon admission because the symptoms had begun three weeks prior, suggesting that more than three days had already elapsed since the event. Administering anticoagulation treatment at that point seemed to be beneficial concerning the stroke. This measure aimed to prevent additional damage, especially since the patient had persistent atrial fibrillation. Her CHA2DS2-VASc score was 6, due to age, female sex, and history of heart failure and hypertension. The HAS-BLED score was 4, due to hypertension, stroke history, age > 65, and medication predisposing to bleeding.

**Figure 1:**
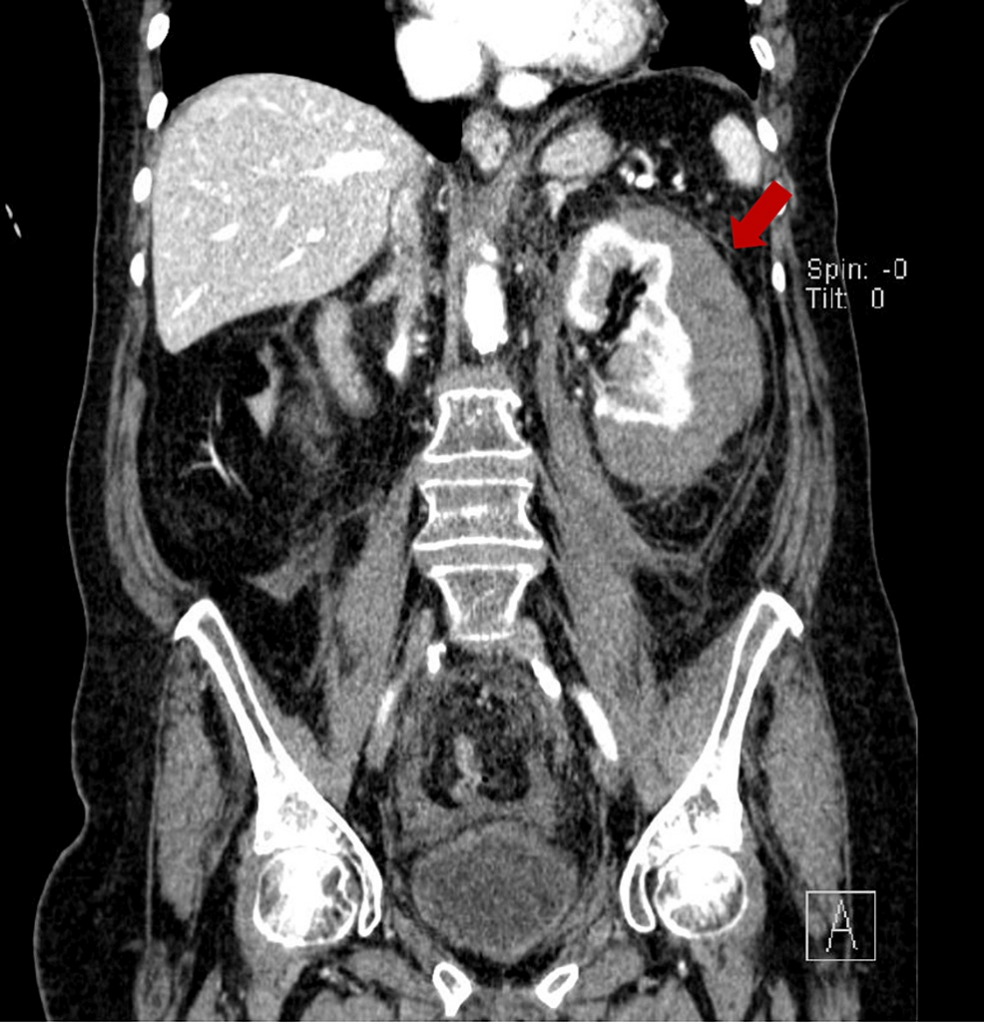
Abdominal computed tomography scan displayed the presence of a perirenal hematoma on the left side, as indicated by the red arrow (coronal view).

**Figure 2:**
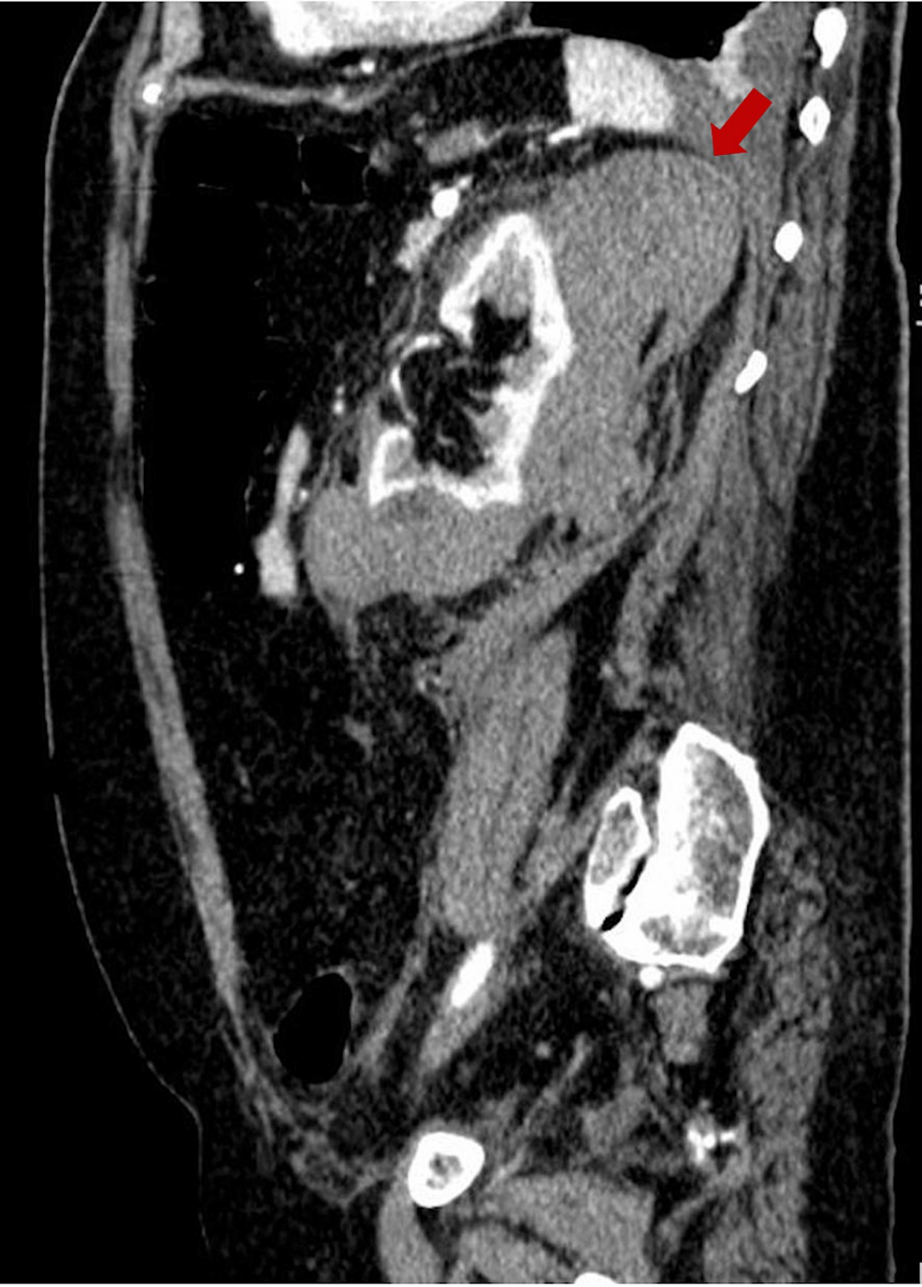
Abdominal computed tomography scan displayed the presence of a perirenal hematoma on the left side, as indicated by the red arrow (sagittal view).

On the fourth day of admission, the patient reported abdominal pain localized in the left iliac fossa. This pain was accompanied by a decrease in hemoglobin levels from 11.3 g/dL to 9.2 g/dL within 24 hours. An abdominal and pelvic contrast-enhanced CT scan was performed, revealing a left perirenal hematoma, hemorrhagic infiltration of the perirenal adipose planes extending along the contour of the left iliac psoas, as well as hemorrhagic infiltration in the adipose planes within the pelvic cavity, predominantly in the presacral region (Figures 1, 2).

The emergency urology team was consulted, and they advised strict bed rest and careful monitoring. Enoxaparin was immediately ceased, and protamine was administered. Transfusional support was unnecessary as the hemoglobin level never dropped below 9.0 g/dL. Additionally, there were no indications of hemodynamic instability.

**Figure 3:**
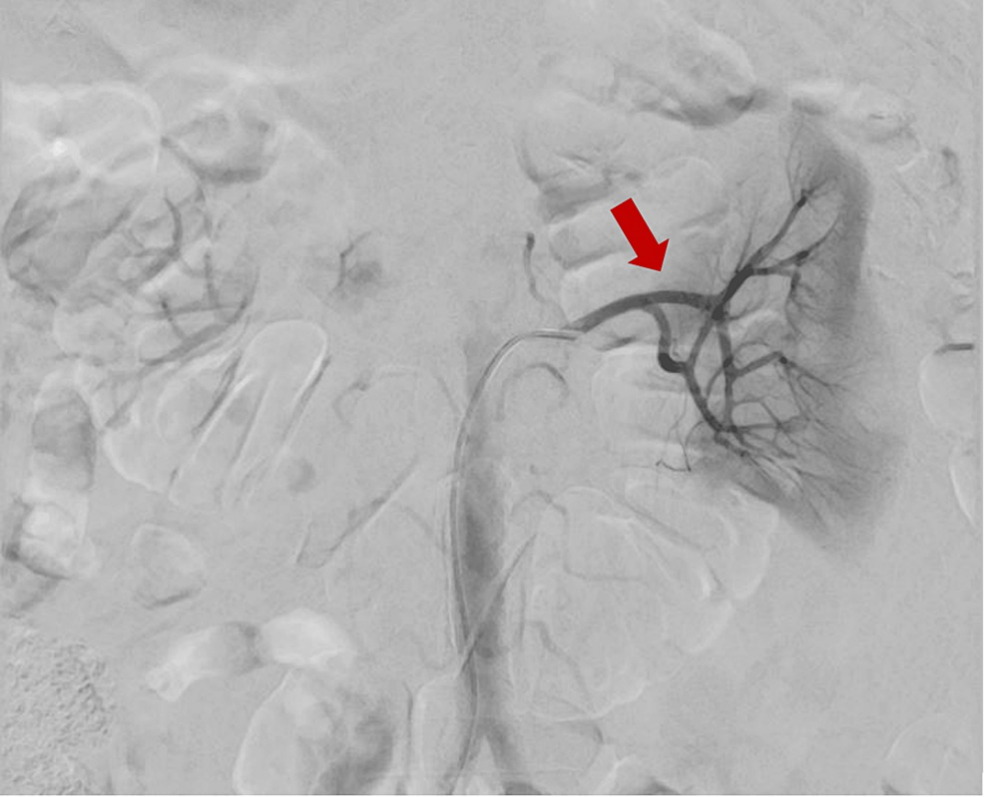
Selective renal arteriography showing the absence of active hemorrhagic foci.

The patient was submitted to selective renal arteriography with right femoral access under ultrasound guidance. Selective catheterization of the left renal artery and renal angiogram showed no evidence of active arterial hemorrhagic extravasations. Multiple supra-selective angiographies were performed targeting the lower pole of the left kidney (where active bleeding was observed in the CT angiography performed approximately 24 hours before). Some subcapsular pathological vessels were identified at times, but no active bleeding was observed at the moment. Given the stable hemodynamic status with normal blood pressure and the absence of active hemorrhagic foci, empirical embolization of the previously bleeding renal territory was not performed (Figure 3).

A conservative approach was chosen, leading to an extended hospital stay. Diagnostic tests were conducted to explore the cause of the stroke, but the conclusion was that it likely resulted from non-adherence with the prescribed medication in a patient with multiple risk factors. Serial CT scans of the abdomen were performed, consistently indicating a decrease in the hematoma size. Notably, there was no acute kidney injury associated with this perirenal hematoma. The patient progressed well, tolerating physiotherapy and achieving a stable mental status without significant neurological deficits, despite showing early signs of dementia.

## Discussion

In this particular case analysis, we depict a patient who experienced a perirenal hematoma associated with enoxaparin usage, which had been prescribed in accordance with current clinical guidelines to prevent the recurrence of cerebral stroke in the context of atrial fibrillation [1]. The development of retroperitoneal hematoma due to subcutaneous enoxaparin anticoagulation therapy is an uncommon complication [2,3]. Risk factors for perirenal hematoma include advanced age, female gender, the use of anticoagulants, and vigorous physical activity [4]. Our patient possessed at least three established risk factors, rendering her highly vulnerable to this bleeding side effect induced by anticoagulant treatment.

In our case, the perinephric hematoma emerged after just 5 doses of enoxaparin. Recent reviews have indicated that in abdominal hematoma cases caused by enoxaparin, the initial symptom or sign of abdominal wall hematoma usually manifests within the initial days after starting the therapy [5].

Physical examination lacks precision in distinguishing abdominal pain in conditions like perinephric hematoma. In this case, CT scans were crucial for diagnosis, although bedside ultrasound would be valuable in the absence of CT scan availability.

Healthcare providers need to be especially vigilant about patients reporting pain, decreased serum hemoglobin levels, or a drop in blood pressure during the initial days of enoxaparin administration, particularly for those at a heightened risk of bleeding. If a hematoma is suspected, diagnostic imaging should be promptly conducted without delay [5].

Initial management requires stopping the anticoagulant medication. Treatment choices are vast. In summary, supportive measures such as pain management, rest, and careful monitoring serve as the initial treatment approach for mild cases of perirenal hematoma. In less severe instances, the body might naturally reabsorb the blood, leading to the gradual resolution of the hematoma over time. Another potential treatment option involves anticoagulation reversal, especially if the perirenal hematoma is caused or exacerbated by anticoagulant medications. In cases where the hematoma is large or actively bleeding, angiography may identify a bleeding vessel and an embolization procedure may be necessary. For severe cases of perirenal hematoma that do not respond to conservative measures, surgical intervention might become necessary (draining the hematoma, repairing damaged blood vessels, or removing clots) [6].

In this case, conservative management was elected, apart from invasive renogram but without the need for arterial embolization. The optimal timing for re-starting anticoagulation remains unclear; in our case, the medical team opted against restarting anticoagulation considering the patient's frailty and dependence. However, upon reviewing the literature, it is evident that further research is necessary to establish the best strategies for the resuming of anticoagulation therapy.

## Conclusions

This study highlighted a rare perinephric hematoma due to enoxaparin use, emphasizing the need for vigilant monitoring. Early diagnosis using CT scans or ultrasound is vital. Management involves stopping anticoagulants and considering interventions like supportive care, anticoagulation reversal, or surgery, tailored to hematoma severity.

Resuming anticoagulation poses a challenge, requiring a personalized approach based on patient factors. Further research is crucial for clear guidelines. Continued vigilance and evidence-based decisions are essential in managing patients on anticoagulants.
